# A meta-analysis of the effects of aerobic exercise on inhibitory control in university students and an analysis of moderating effects

**DOI:** 10.3389/fpsyg.2026.1992560

**Published:** 2026-10-29

**Authors:** Shaofeng Peng, Pinhong Wu, Qingfang Zeng, Lixia Liu, XinJie Pan, Shen Wang, Haiqing Feng

**Affiliations:** 1 School of Physical Education and Sport Science, Fujian Normal University, Fuzhou, China; 2 College of Physical Education and Sports, Beijing Normal University, Beijing, China; 3 The 910 Hospital of the PLA Joint Logistic Support Force, Quanzhou, China; 4 Fujian Provincial Institute of Sports Science, Fuzhou, China

**Keywords:** aerobic exercise, executive function, inhibitory control, Meta-analysis, moderator analysis, randomized controlled trial, university students

## Abstract

**Background:**

Aerobic exercise has been increasingly recognized as a potential strategy for enhancing executive function, yet its effect on inhibitory control in university students remains inconsistent, and the intervention characteristics associated with effect variability are unclear. This meta-analysis aimed to synthesize randomized controlled trial evidence on the effects of aerobic exercise on inhibitory control in university students and to explore potential moderators.

**Methods:**

PubMed, PsycARTICLES, PsycINFO, ScienceDirect, Web of Science, Cochrane Library, CNKI, Wanfang, and VIP were systematically searched from inception to March 2026. Randomized controlled trials involving university students aged 18–25 years, aerobic exercise interventions, eligible control conditions, and validated inhibitory-control tasks were included. Hedges’ g was used as the effect-size metric. Random-effects models with restricted maximum likelihood estimation and Hartung–Knapp adjustment were applied. Subgroup analyses examined participant type, intervention period, exercise intensity, exercise session duration, and task paradigm, and exploratory univariable multilevel meta-regression was conducted to account for multiple effects nested within studies.

**Results:**

Nine independent studies involving 605 participants were included. Aerobic exercise produced a small but statistically significant improvement in inhibitory control (Hedges’ *g* = −0.353, 95% CI [−0.591, −0.115], *p* = 0.009), with low statistical heterogeneity (*I*^2^ = 26.98%). Leave-one-study-out analyses showed stable pooled estimates (*g* = −0.397 to −0.288). No statistically significant between-group differences were observed for participant type, intervention period, exercise intensity, exercise session duration, or task paradigm. Exploratory meta-regression likewise identified no statistically significant moderator associations. Egger’s test was not significant (*p* = 0.072).

**Conclusion:**

Aerobic exercise may produce a small but statistically significant improvement in inhibitory control among university students; however, the practical significance of this effect remains uncertain, and the current evidence is entirely based on studies conducted in China. Although some intervention subgroups showed numerically different effect estimates, the limited number of independent studies means that the current evidence is insufficient to determine whether participant or exercise characteristics moderate the intervention effect. Further high-quality randomized trials are needed to clarify dose–response relationships and optimize aerobic exercise prescriptions for inhibitory-control enhancement.

**Systematic Review Registration:**

https://www.crd.york.ac.uk/PROSPERO/view/CRD420261335563. CRD420261335563).

## 1 Introduction

In a complex information environment, an individual’s capacity for self-control is particularly crucial. Inhibitory control, a key component of executive function, refers to an individual’s ability to suppress dominant responses, resist distracting stimuli, and regulate their behaviour, cognition and emotional responses in accordance with goal requirements (Diamond, 2013; Lee et al., 2025; Sosa, 2024). Executive function, as a vital higher-order cognitive process that underpins goal-directed behaviour, comprises three core components: inhibitory control, working memory and cognitive flexibility. It plays a crucial role in information processing, behavioural regulation and decision-making within complex environments (Badre, 2025; Diamond, 2013; Tervo-Clemmens et al., 2023; Yanguez et al., 2024). As a key demographic in the stage of ongoing cognitive development and the shaping of behavioural patterns, university students need to rely on sound inhibitory control to regulate emotional and behavioural responses, resist immediate temptations, and maintain goal-directed behaviour and the pursuit of long-term goals (Inzlicht et al., 2021; Luciana and Collins, 2022; Tervo-Clemmens et al., 2023; Werner and Berkman, 2024). However, as the information environment becomes more complex, academic pressures increase and lifestyles change, the cognitive control abilities of university students are receiving increasing attention (Belluardo et al., 2025; Lin et al., 2020; McGrath et al., 2023). Therefore, exploring effective strategies to enhance inhibitory control in university students is of great significance for optimising cognitive performance and promoting mental health.

As a safe, cost-effective and easily implementable non-pharmacological intervention, aerobic exercise has attracted widespread attention in recent years for its role in enhancing cognitive function (Chang et al., 2025; Ishihara et al., 2021; Singh et al., 2025; Ye et al., 2024). Existing research suggests that aerobic exercise may influence higher-order cognitive processing through mechanisms such as increasing cerebral blood flow, promoting the release of neurotrophic factors, and modulating prefrontal cortex-related neural activity (Byun et al., 2014; Moreau and Chapple-De Lange, 2026; Tomoto et al., 2023). Previous research has shown that the cognitive effects of exercise are not limited to the domain of executive function; previous systematic reviews and meta-analyses suggest that exercise may also influence memory function, and that the effects of acute exercise on episodic memory may be influenced by factors such as the temporal relationship between exercise and memory tasks (Loprinzi et al., 2019; Loprinzi et al., 2018). In particular, inhibitory control relies on the prefrontal cortex and associated executive control networks, and the relationship between these functions and exercise-induced physiological and neural changes has also attracted considerable attention (Friedman and Robbins, 2022; Ishihara et al., 2021). Consequently, the impact of aerobic exercise on inhibitory control has gradually attracted attention in the field of exercise cognition.

In recent years, a large number of experimental studies have examined the effects of aerobic exercise on inhibitory control in university students, using classic cognitive paradigms such as the Stroop task, the Flanker task and the Go/No-go task (Ishihara et al., 2021; Li et al., 2025; Remiszewski et al., 2025). Some studies have found that acute aerobic exercise can enhance an individual’s efficiency in processing conflicting information, as evidenced by shorter reaction times or lower error rates (Chang et al., 2025; Kujach et al., 2018). Long-term aerobic exercise interventions have also been found to potentially improve an individual’s response inhibition and interference suppression abilities. For example, several studies involving acute exercise trials at different intensities have found that moderate-intensity aerobic exercise may produce a more pronounced enhancement of inhibitory control than low- or high-intensity exercise, suggesting that exercise intensity may influence the relationship between physical activity and cognitive performance (Kujach et al., 2018). Furthermore, long-term exercise intervention studies have also shown that regular aerobic exercise may improve inhibitory control in specific groups, such as university students with internet or smartphone addiction (Nie and Fan, 2025; Wang and Li, 2025). However, not all studies have observed consistent positive effects; some studies have found that performance on inhibitory tasks did not show significant improvement following aerobic exercise interventions (Ciria et al., 2018; Ishihara et al., 2021; Kao et al., 2022).

These inconsistent findings suggest that the effects of aerobic exercise on inhibitory control may exhibit some heterogeneity. The underlying causes of these differences may be related to the characteristics of the exercise interventions and the characteristics of the participants (Ishihara et al., 2021; Ludyga et al., 2020; Zhang et al., 2026). On the one hand, aerobic exercise is not a single form of intervention; its effects may be influenced by factors such as exercise intensity, type of exercise, duration of the intervention, exercise session duration, and total exercise volume. On the other hand, individual differences—such as age, gender, baseline physical activity levels, and cognitive status—may also influence the cognitive adaptation process following exercise intervention. However, existing research has largely focused on testing effects under single experimental conditions, and there remains a lack of systematic analysis regarding how different intervention parameters and individual characteristics modulate the impact of aerobic exercise on inhibitory control.

To date, meta-analyses have examined the relationship between exercise and executive function from various perspectives. For example, Ishihara et al. (2021) conducted a meta-analysis of individual participant data on the effects of acute aerobic exercise on executive function, whilst Dkaidek et al. (2023) further investigated the impact of a single session of power cycling on executive function in young adults. Recently, Zhang et al. (2026) conducted a systematic review and meta-analysis of exercise interventions and executive function in university students, reporting effects on inhibitory control, working memory and cognitive flexibility, respectively. However, there remain significant differences between existing meta-analyses and the research focus of this study. Ishihara et al. focused primarily on a single session of acute aerobic exercise and were therefore unable to address the effects of long-term aerobic training on inhibitory control (Ishihara et al., 2021; Dkaidek et al., 2023) further restricted the intervention to a single session of high-power cycling, and their conclusions are likewise primarily applicable to acute exercise scenarios. Although Zhang et al. included both acute and long-term exercise and reported the effects on inhibitory control separately, their overall research framework encompassed a variety of exercise forms—including aerobic exercise, high-intensity interval training, resistance training, Tai Chi, jumping and Tabata—whilst examining three components of executive function: inhibitory control, working memory and cognitive flexibility. Consequently, their analysis of the specific relationship between “aerobic exercise and inhibitory control” remains primarily a component of a broader exercise–executive function framework (Zhang et al., 2026). Furthermore, Zhang et al. (2026) examination of factors such as exercise type, intensity, session duration and intervention period was conducted primarily through subgroup analyses, without further employing meta-regression to test the associations between these potential moderators and the effect sizes. It is therefore still necessary to further restrict the intervention type to aerobic exercise and the outcome to inhibitory control, and, based on a unified definition of effect size, to further examine the relationship between exercise intensity, intervention period, exercise session duration, participant type and task paradigm, and differences in intervention effects.

On this basis, this study further focuses on aerobic exercise as a specific form of intervention and inhibitory control as a specific component of executive function. It includes only randomised controlled trials and uses meta-analysis to comprehensively evaluate the overall effect of aerobic exercise on inhibitory control in university students. Furthermore, methods such as exploratory subgroup analysis and univariate multilevel meta-regression are employed to examine the relationship between exercise intensity, intervention period, exercise session duration, participant type and task paradigm with variations in the observed effects. The aim is to provide more targeted quantitative evidence building on existing research into exercise and executive function, and to offer guidance for the development of cognitive enhancement programmes and the implementation of exercise interventions for university students.

## 2 Methods

This study conducted its literature search in strict accordance with the Cochrane Handbook for Systematic Reviews and Meta-Analyses and the PRISMA (Preferred Reporting Items for Systematic Reviews and Meta-Analyses) guidelines (registration number: CRD420261335563).

### 2.1 Literature search

A search was conducted for Chinese and English literature published both domestically and internationally, covering the period from the inception of each database to March 2026. The literature search employed a combination of subject headings and free-text terms; the English search terms primarily included terms related to the study population, such as “students” OR “young adult” OR “college student” OR “university student” OR “undergraduate,” terms related to the intervention, such as “exercise” OR “aerobic exercise” OR “aerobic” OR “endurance” OR “running” OR “cycling” OR “swimming” OR “walking,” and terms related to outcome measures, such as “cognition” OR “executive function” OR “inhibitory control.” Searches were conducted in foreign-language databases such as PubMed, PsycARTICLES, PsycINFO, ScienceDirect, Web of Science and the Cochrane Library. Chinese literature was primarily retrieved from CNKI, Wanfang Database and VIP Database, using search terms including “students OR university students OR young people OR undergraduates OR higher education students,” “aerobic exercise OR aerobic training OR exercise intervention OR endurance training OR cycling OR jogging OR walking OR swimming,” and “cognitive function OR executive function OR inhibitory control.” Additionally, a back-search was conducted on the references of the included studies and relevant reviews to enhance the comprehensiveness of the literature search. The initial search was conducted in January 2026, with an updated search carried out in March 2026.

### 2.2 Inclusion and exclusion criteria

#### 2.2.1 Inclusion criteria

1. Population: The study included university students aged between 18 and 25, regardless of gender. The age range was restricted to 18–25 in order to focus on young university students at a relatively consistent stage of development and to minimise the heterogeneity in developmental stages that a wider age range might entail; this age range corresponds to the classic core age range of ‘emerging adulthood’ (Arnett, 2000).
2. Intervention: The experimental group receives an aerobic exercise intervention, comprising typical forms of aerobic exercise such as running, cycling, swimming and walking; the study must clearly report relevant information such as exercise intensity, duration of each session, frequency of exercise and duration of the intervention.
3. Comparison: The control group receives no exercise intervention, engages in routine physical activity or is placed on a waiting list.
4. Outcome Measures: Assessment must be based on at least one classic inhibitory control task, including the Stroop task, the Flanker task or the Go/No-go task; data metrics suitable for calculating effect sizes (such as mean, standard deviation and sample size) must be reported.
5. Study Design: Randomised controlled trials (RCTs) are included.

#### 2.2.2 Exclusion criteria

1. Reviews, meta-analyses, animal studies, case reports, cross-sectional studies, cohort studies and non-randomised experimental studies.
2. Studies in which the study population did not consist of university students, or from which data on university students could not be extracted separately.
3. Studies with incomplete data from which effect sizes could not be calculated.
4. Duplicate publications.
5. Literature for which the full text could not be obtained or from which valid research data could not be extracted.

### 2.3 Selection of literature and data extraction

The retrieved literature was first de-duplicated using literature management software; subsequently, two researchers independently carried out title and abstract screening, full-text assessment, data extraction and coding in accordance with pre-defined inclusion and exclusion criteria. Where discrepancies arose, these were resolved through discussion or by consulting a third-party researcher until consensus was reached (Guimarães et al., 2022; Page et al., 2021). The data extracted from the studies primarily included the first author, year of publication, study location, sample size, basic characteristics of the study participants, exercise intervention protocol (type of exercise, exercise intensity, exercise frequency, duration of each session and duration of the intervention), control conditions, type of inhibitory control task and relevant statistical data. The specific coding details are shown in Table 1.

**Table 1:** Basic characteristics of the included studies on the effects of aerobic exercise on inhibitory control in university students.

Study (year)	Region	Study design	Sample size	Participants/Age (years)	Aerobic intervention	Exercise intensity	Dose (frequency/session duration/period)	Control condition	Inhibitory-control task and outcome metric	Risk of bias
Yang and He (2023)	China	RCT	60 (20/20/20)	General university students; 20 ± 2	Swimming (HIIT/MICT)	HIIT: ≥85% HRmax; MICT: 60–80% HRmax	4 sessions/week; 30 min/session; 12 weeks	No swimming intervention	Flanker; interference score (incongruent − congruent)	Some concerns
Zhang et al. (2017)	China	RCT	52 (26/26)	Female university students; 19.36 ± 1.17	Aerobic running	60–69% HRmax	3 sessions/week; 40 min/session; 8 weeks	Normal study and daily life	Flanker; interference score (incongruent − congruent)	Some concerns
Yu (2023a)	China	RCT	60 (20/20/20)	Overweight university students (special population); 20.57 ± 1.12	Cycle ergometer	75–85% HRmax; 65–75% HRmax	Single session; 20 min	Control group (details not further specified)	Flanker; incongruent-trial reaction time	Some concerns
Yu (2023b)	China	RCT	60 (20/20/20)	University students with smartphone addiction (special population); not reported	Aerobic exercise	75–85% HRmax; 65–75% HRmax	3 sessions/week; 45 min/session; 8 weeks	Control group (details not further specified)	Stroop; incongruent-trial reaction time	Some concerns
Li et al. (2020)	China	RCT	40 (21/19)	Female university students; 18.5 ± 0.5	Aerobic exercise	60–69% HRmax	3 sessions/week; 45 min/session; 8 weeks	Maintained normal life and study	Stroop; incongruent-trial reaction time	Some concerns
Nie and Fan (2025)	China	RCT	120 (30/30/30/30)	University students with internet addiction (special population); 21.0 ± 1.0	Cycling, basketball, and swimming	55–65% HRmax	2 sessions/week; 45 min/session; 8 weeks	No additional exercise; usual lifestyle maintained	Flanker; interference score (incongruent − congruent)	Some concerns
Gao et al. (2025)	China	RCT	81 (27/26/28)	University students with depressive symptoms (special population); 19.61 ± 0.96	Aerobic running and taekwondo	Moderate intensity: 64–76% HRmax (main exercise phase)	Single session; 30 min	Non-intervention control	Stroop; incongruent-trial reaction time	High risk
Wang et al. (2023)	China	RCT	81 (27/26/28)	General university students; 22.4 ± 2.5	Cycle-ergometer HIIT	High intensity (monitored using Borg RPE)	1 or 2 sessions/week; 20 min/session; 6 weeks	No HIIT; normal living conditions maintained	Stroop; incongruent-trial reaction time	High risk
Si et al. (2025)	China	RCT	51 (17/17/17)	Male university students; 21.06 ± 1.6	Cycle ergometer (VIIT/MICT)	VIIT high-intensity phase: 75–85% HRR; MICT: 40–60% HRR	3 sessions/week; 33–36 min/session; 6 weeks	Watched a video for 30 min	Stroop; overall indicator	Some concerns

RCT, randomized controlled trial. Numbers in parentheses indicate group sample sizes. All studies included pre- and post-intervention assessments; Wang et al. (2023) additionally reported a follow-up assessment 6 weeks after the intervention. Exercise intensity is presented as reported in the original studies; for multi-arm trials, all eligible aerobic-exercise intervention arms included in the analysis are shown. HRmax, maximal heart rate; HRR, heart-rate reserve; HIIT, high-intensity interval training; MICT, moderate-intensity continuous training; VIIT, variable-intensity interval training; RPE, rating of perceived exertion. The specific inhibitory-control outcome metrics are aligned with those used for effect-size extraction in Supplementary Table S1; where the original report did not further specify the control condition, this is stated explicitly.

### 2.4 Assessment of the quality of the included studies

Two researchers assessed the included studies using the Cochrane Risk of Bias 2 (RoB 2) tool, which covers five domains: the randomisation process, deviation from the intended intervention, missing outcome data, outcome measurement, and selective reporting of results; the overall risk of bias was categorised as “Low risk,” “Some concerns” or “High risk” (Sterne et al., 2019).

### 2.5 Calculation of the effect size

This study employed Hedges’ g, calculated as the standardised mean difference, as the effect size measure to reduce bias in effect size estimates under small-sample conditions (Nakamura, 2026; Suero et al., 2023). Effect sizes were calculated using Comprehensive Meta-Analysis Version 3.3 (CMA 3.3) software. For randomised controlled trials employing a pre-test–post-test design, the primary analysis calculated the standardised mean difference between the intervention and control groups based on the post-test means, standard deviations and sample sizes at the end of the intervention, and further applied small-sample correction to obtain Hedges’ g. To ensure consistency in the definition of effect sizes across different studies, the primary analysis employed a uniform post-test between-group comparison method, rather than a mixed approach using change scores or effect estimates adjusted for covariates. Baseline data were primarily used to assess the comparability of the experimental and control groups prior to the intervention and were not directly included in the calculation of the primary effect size.

This study draws upon Cohen’s classic criteria for evaluating effect sizes, using 0.2, 0.5 and 0.8 as the thresholds for small, medium and large effect sizes, respectively, (Andrade, 2020). With regard to the coding of effect size direction, the direction of performance improvement was first determined based on the definitions of each outcome measure in the original study. The inhibitory control measures used in the main analysis were all reaction times or conflict/interference measures calculated from reaction times. For measures where a decrease in score indicates improved inhibitory control performance, the effect size was calculated based on the raw between-group difference between the experimental and control groups; if the scoring direction of the original measure was opposite to the unified effect direction, the sign of the effect size was reversed. Once the direction was unified, a value of Hedges’ *g* < 0 indicated that the aerobic exercise group performed better in inhibitory control than the control group, whilst a value of Hedges’ *g* > 0 indicated that the control group performed better. Overall and subgroup effects were analysed using a random-effects model with REML estimation, and 95% confidence intervals and significance levels were calculated using the Hartung–Knapp method (Langan et al., 2019; van Aert and Jackson, 2019). Heterogeneity between studies was assessed using Cochran’s *Q* statistic, the *I*^2^ statistic and the estimate of between-study variance (τ^2^), with τ^2^ estimated using the REML method. It is generally accepted that *I*^2^ values of 25%, 50 and 75% represent low, moderate and high levels of heterogeneity, respectively (Cordero and Dans, 2021).

For multi-arm randomised controlled trials comprising multiple intervention arms that meet the inclusion criteria and share the same control group, this study was conducted in accordance with the methodological recommendations for Cochrane systematic reviews (Rücker et al., 2017). Where multiple intervention arms are clinically and methodologically homogeneous, they are combined into a single intervention arm for comparison with the shared control arm; the pooled sample size is the sum of the sample sizes of the individual intervention arms, the pooled mean is calculated using sample size weighting, and the pooled standard deviation is calculated using the formula recommended in the Cochrane Handbook. For moderator analyses requiring the retention of information from different intervention arms—such as those concerning exercise intensity—the individual intervention arms are retained, and the sample size of the shared control group is split according to the number of included intervention arms, whilst the mean and standard deviation of the control group remain unchanged (Rücker et al., 2017). This procedure is used to avoid repeated weighting of the same control participants and the resulting unit-of-analysis problem; however, it does not make the resulting effect-size comparisons statistically independent. In multilevel meta-regression analyses, multiple effect sizes from the same study are therefore nested within the study to account for their within-study dependence (Fernández-Castilla et al., 2020). In the analysis of overall effects, multiple intervention arms that are clinically and methodologically homogeneous are pooled to avoid double-counting of the shared control group; however, for moderating analyses—such as those involving exercise intensity—where information on the different intervention arms needs to be retained, each arm is retained separately to examine the relationship between different intervention characteristics and the effect size. Details of the specific outcome measures, post-test data and information on effect size calculations for each study included in the primary analysis are set out in Supplementary Table S1.

### 2.6 Assessment of publication bias and sensitivity analysis

A preliminary assessment of potential publication bias was conducted using funnel plots and the fail-safe number (Nfs), and publication bias was further tested using Egger’s linear regression method (Sterne et al., 2011). At the same time, sensitivity analyses were conducted by sequentially excluding individual studies to examine the impact of each study on the overall effect size and the stability of the study conclusions (Kunze et al., 2023). In addition, sensitivity analyses were conducted on studies assessed as high risk for overall bias to examine the impact of study quality on the estimates of the overall effect.

### 2.7 Analysis of moderating factors

To further investigate the potential sources of heterogeneity in the effects of aerobic exercise interventions, this study, drawing on existing theoretical foundations and a pre-defined coding scheme, identified participant type, intervention period, exercise intensity, exercise session duration and task paradigm as potential moderator variables (Page et al., 2021). In this study, participants were categorised into the general population and specific groups; the specific groups comprised a sample of university students specifically recruited on the basis of characteristics such as overweight, internet or smartphone addiction, or symptoms of depression. The intervention period was classified according to duration into single acute exercise sessions and long-term exercise training programmes lasting several weeks. In this study, the exercise session duration refers to the actual duration of each training session or single acute exercise session, as distinct from the intervention period, which denotes the overall duration of the intervention. Given the differences between the two types of intervention in terms of time scale and potential mechanisms of action, in addition to the overall combined analysis, a sensitivity analysis was conducted that included only studies on long-term exercise training to examine whether the overall effect estimate was influenced by studies on acute exercise.

Exercise intensity was standardised using a hierarchical coding principle. Firstly, if the original study explicitly reported that the intervention was of moderate or high intensity, the intensity classification from the original study was prioritised; if the original study did not explicitly specify the intensity category, coding was performed based on the reported physiological or subjective load indicators, with reference to the relative exercise intensity standards of the American College of Sports Medicine (ACSM). Moderate intensity is typically defined as 40–59% of heart rate reserve (HRR) or oxygen consumption reserve (VO₂R), 64–76% of maximum heart rate (HRmax), 46–63% of maximum oxygen consumption (VO₂max), or a Borg 6–20 scale RPE of 12–13; High intensity is typically defined as 60–89% of HRR or VO₂R, 77–95% of HRmax, 64–90% of VO₂max, or an RPE of 14–17 on the Borg scale; For studies reporting target intensity ranges that span adjacent categories or employ variable-intensity or interval training protocols, coding is based on the original study’s definition of intervention intensity and the target intensity for the main exercise phases (Garber et al., 2011; Huang et al., 2026).

The exercise session duration was categorised into two groups— ≤30 min and >30 min—using a 30-min threshold, based on the stratification method employed in previous systematic reviews and meta-analyses examining the association between exercise and executive function (Zhang et al., 2026).

For categorical variables, subgroup analyses were conducted to compare differences in effect sizes across different study characteristics. Given the limited number of included independent studies, and to reduce the risk of overfitting and unstable regression coefficients in multivariate models, the meta-regression employed an exploratory univariate analysis strategy. Specifically, independent multilevel random-effects meta-regression models were established separately for participant type, intervention period, exercise intensity, exercise session duration, and task paradigm, rather than including multiple moderator variables simultaneously within a single model (Higgins and Thompson, 2004). Where a single study contributed multiple effect sizes, these were nested within the study to account for the interdependence of effect sizes within that study (Fernández-Castilla et al., 2020). Given the limited number of independent studies, all meta-regression results are interpreted as exploratory findings.

## 3 Results

### 3.1 Inclusion and coding of literature

The literature screening process is illustrated in Figure 1. Following database searches, literature screening and full-text evaluation, nine articles meeting the inclusion criteria were ultimately included. These comprised nine independent studies with a total sample size of 605 participants (Gao et al., 2025; Li et al., 2020; Nie and Fan, 2025; Si et al., 2025; Wang et al., 2023; Yang and He, 2023; Yu, 2023a, 2023b; Zhang et al., 2017). Of these, nine studies were conducted in China and none abroad. All included studies employed a randomised controlled trial design, with the study participants primarily comprising university students aged 18–25. The types of studies included acute aerobic exercise interventions and long-term exercise interventions. Based on participant characteristics, the included studies comprised both general university student populations and university student populations with specific characteristics. Among these, four studies involving special populations, totalling 321 participants, included overweight university students, university students with internet addiction, and university students with depressive symptoms; five studies involving general university students, totalling 284 participants, were also included. Interventions primarily comprised swimming, running, stationary cycling, aerobics and comprehensive aerobic exercise programmes, whilst the assessment tasks mainly included the Stroop task and the Flanker task. One study reported follow-up measurements, with a follow-up period exceeding 6 weeks. Quality assessment was conducted using the Cochrane Risk of Bias Tool 2.0. Among the included studies, none were classified as having a low risk of bias; seven were classified as having some concerns; and two were classified as having a high risk of bias. The specific results of the risk of bias assessment are shown in Figure 2, whilst the basic characteristics and coding information of the included studies are presented in Table 1.

**Figure 1:**
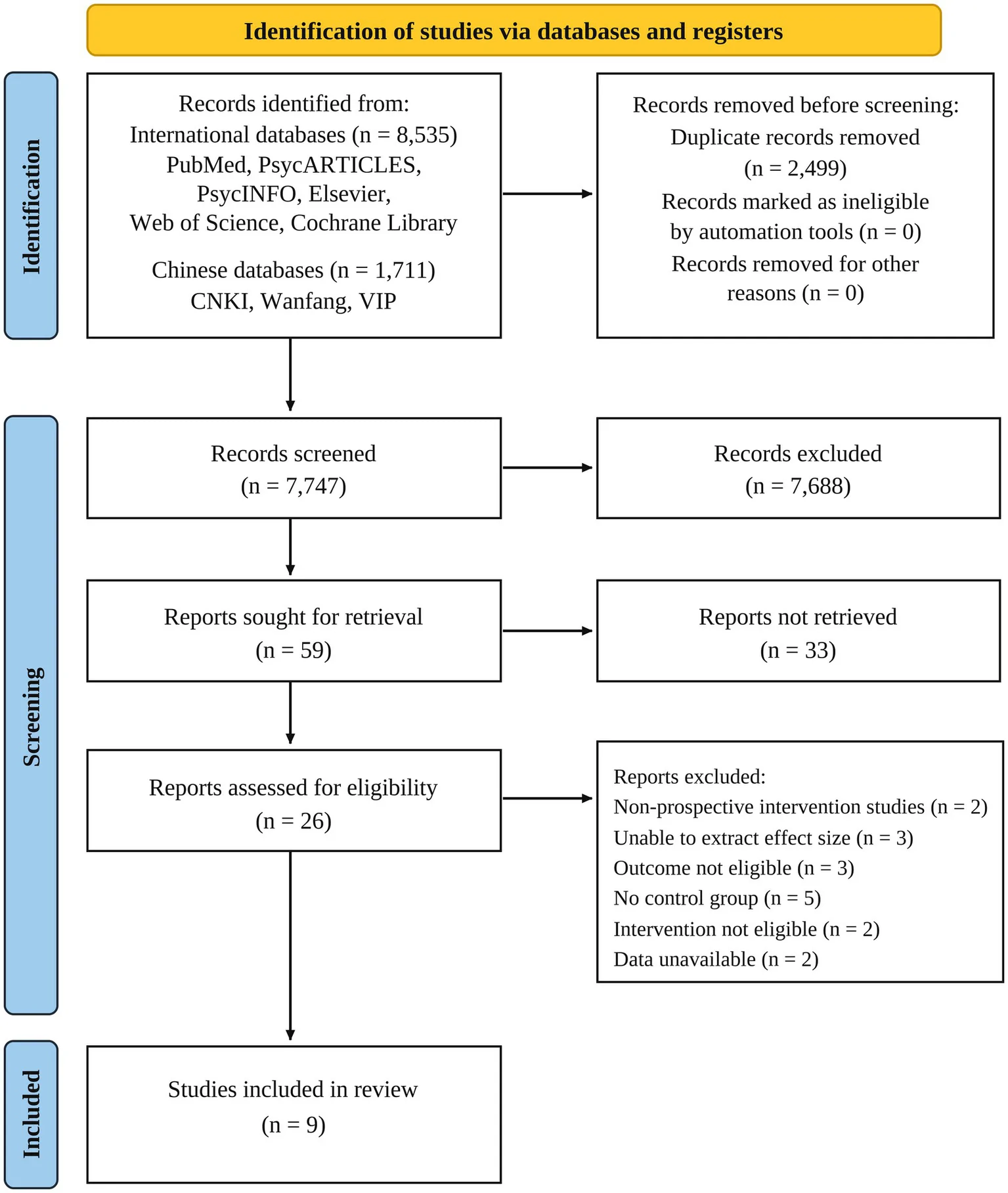
PRISMA flow diagram of the literature search and study selection process.

**Figure 2:**
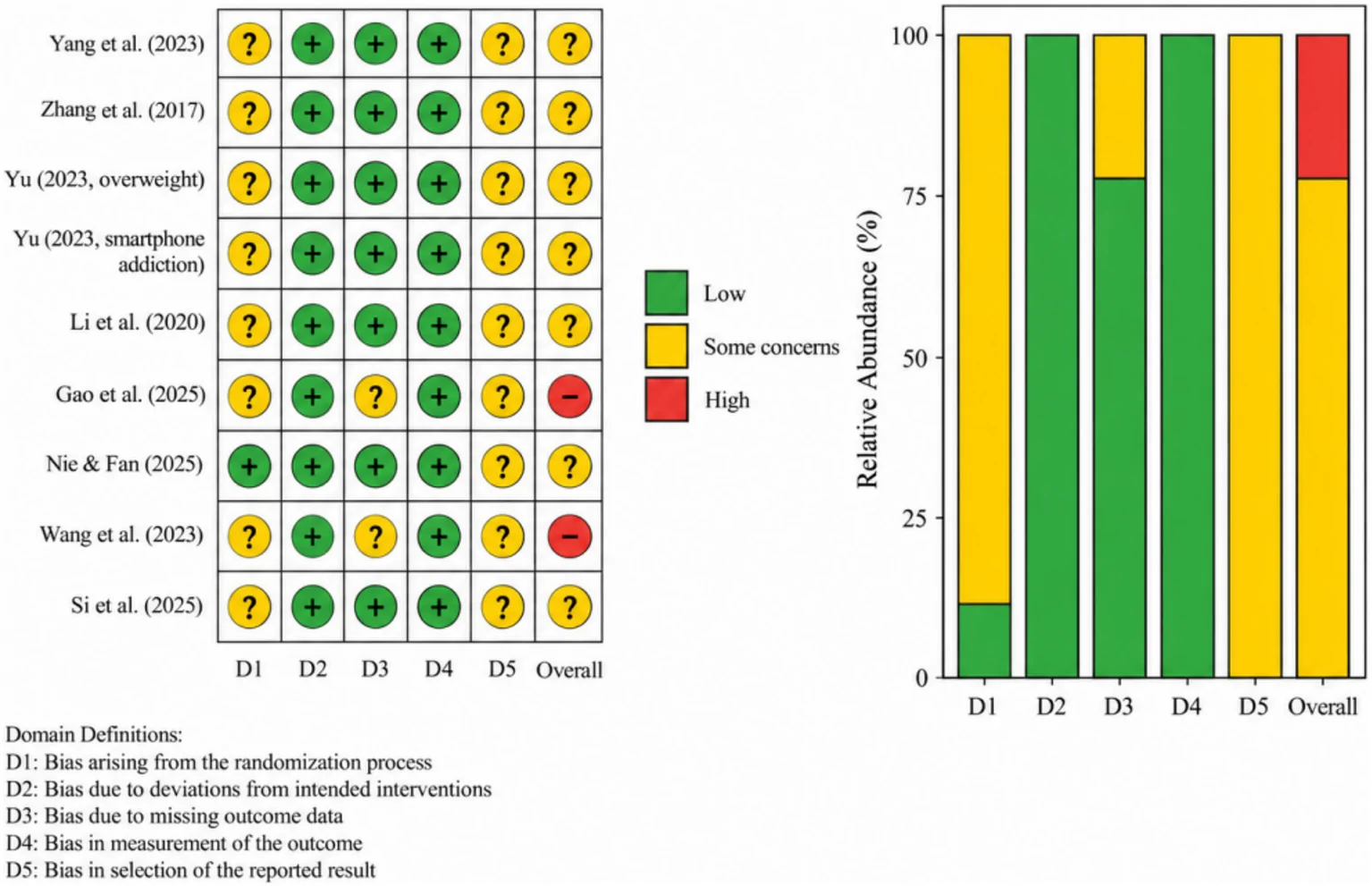
Risk-of-bias assessment of the included studies using the Cochrane RoB 2 tool. Note: D1, bias arising from the randomization process; D2, bias due to deviations from intended interventions; D3, bias due to missing outcome data; D4, bias in measurement of the outcome; D5, bias in selection of the reported result. Green indicates low risk, yellow indicates some concerns, and red indicates high risk.

### 3.2 The effect of aerobic exercise on inhibitory control in university students

The nine included studies comprised a total of nine independent research projects. A meta-analysis using a random-effects model was conducted to assess the overall effect of aerobic exercise interventions on inhibitory control in university students. The results indicated that aerobic exercise significantly improved inhibitory control in university students (*g* = −0.353, 95% CI: −0.591 to −0.115, *p* = 0.009), with the overall effect size reaching the level of a small effect. As the direction of the effect size was standardised in this study—with a negative value indicating that the exercise intervention group performed better in terms of inhibitory control than the control group—these results suggest that aerobic exercise can, to a certain extent, improve inhibitory control performance in university students. The results of the heterogeneity test indicated that there was low statistical heterogeneity in the effect sizes (*Q* = 10.96, df = 8, *I*^2^ = 26.98 per cent, τ^2^ = 0.012). Further sensitivity analysis revealed that, upon sequentially excluding individual studies, the pooled effect size ranged from *g* = −0.397 to −0.288; the direction of the effect remained unchanged in all cases and was statistically significant in each instance, indicating that the overall results are reasonably robust. Furthermore, given the differences in intervention period between single-session acute exercise and long-term exercise training, two studies involving single-session acute exercise were further excluded, and only seven long-term exercise training studies were included in the sensitivity analysis. The results showed that the pooled effect in the sensitivity analysis restricted to long-term training was *g* = −0.331 (95% CI: −0.662 to 0.001, *p* = 0.050), with between-study heterogeneity of *I*^2^ = 40.93% (Q = 10.16, τ^2^ = 0.041). The direction and magnitude of this effect were similar to those of the overall analysis, although the 95% confidence interval straddled 0 slightly. After further excluding two studies with a high overall risk of bias, the pooled effect of the remaining seven studies was *g* = −0.383 (95% CI: −0.729 to −0.037, *p* = 0.035), with *I*^2^ = 43.55% and τ^2^ = 0.053; the direction and magnitude of the effect were essentially consistent with those of the overall analysis. The results of the publication bias test showed that the Egger linear regression test did not reach statistical significance (*p* = 0.072), with a fail-safe number (Nfs) of 138; however, given the small number of included studies, the statistical power of the Egger test is limited, and therefore the possibility of publication bias or the small-sample effect cannot be ruled out on this basis. The specific results are shown in Table 2, Figure 3 and Figure 4.

**Table 2:** Overall effect, heterogeneity, sensitivity analysis, and publication bias of aerobic exercise on inhibitory control.

Outcome	*k*	*N*	*g* (95% CI)	Sensitivity analysis	Heterogeneity test				Publication bias test				
				*g* (95% CI)	*Q*_w_	*df*	*I* ^2^	τ^2^	*N*_fs_	Egger’s intercept	SE	(95% CI)	*p*
Inhibitory control	9	605	−0.353 (−0.591, −0.115)	No outliers	10.96	8	26.98	0.012	138	−4.83	2.50	(−10.147, 0.490)	0.072

k, number of independent studies; *N*, total sample size; g, Hedges’ g; CI, confidence interval; Qw, Cochran’s heterogeneity statistic; df, degrees of freedom; *I*^2^, percentage of total variability attributable to between-study heterogeneity;τ^2^, between-study variance estimated using the restricted maximum likelihood (REML) method. Nfs, fail-safe number; SE, standard error. Negative Hedges’ *g* values indicate better inhibitory control performance in the aerobic exercise group than in the control group.

**Figure 3:**
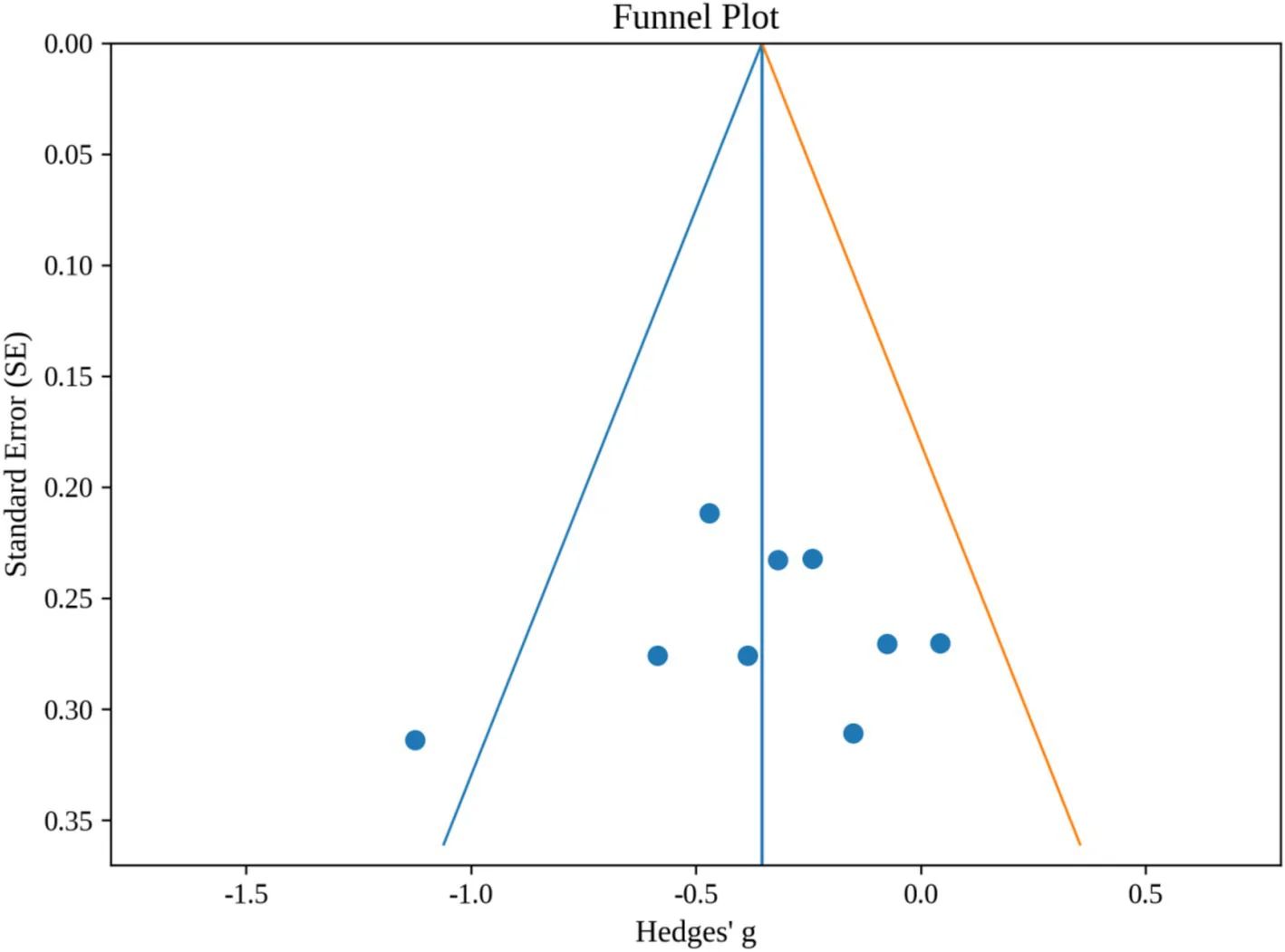
Funnel plot of the overall effect of aerobic exercise on inhibitory control in university students. Note: Each point represents one independent study-level effect size. The vertical line indicates the pooled effect estimate.

**Figure 4:**
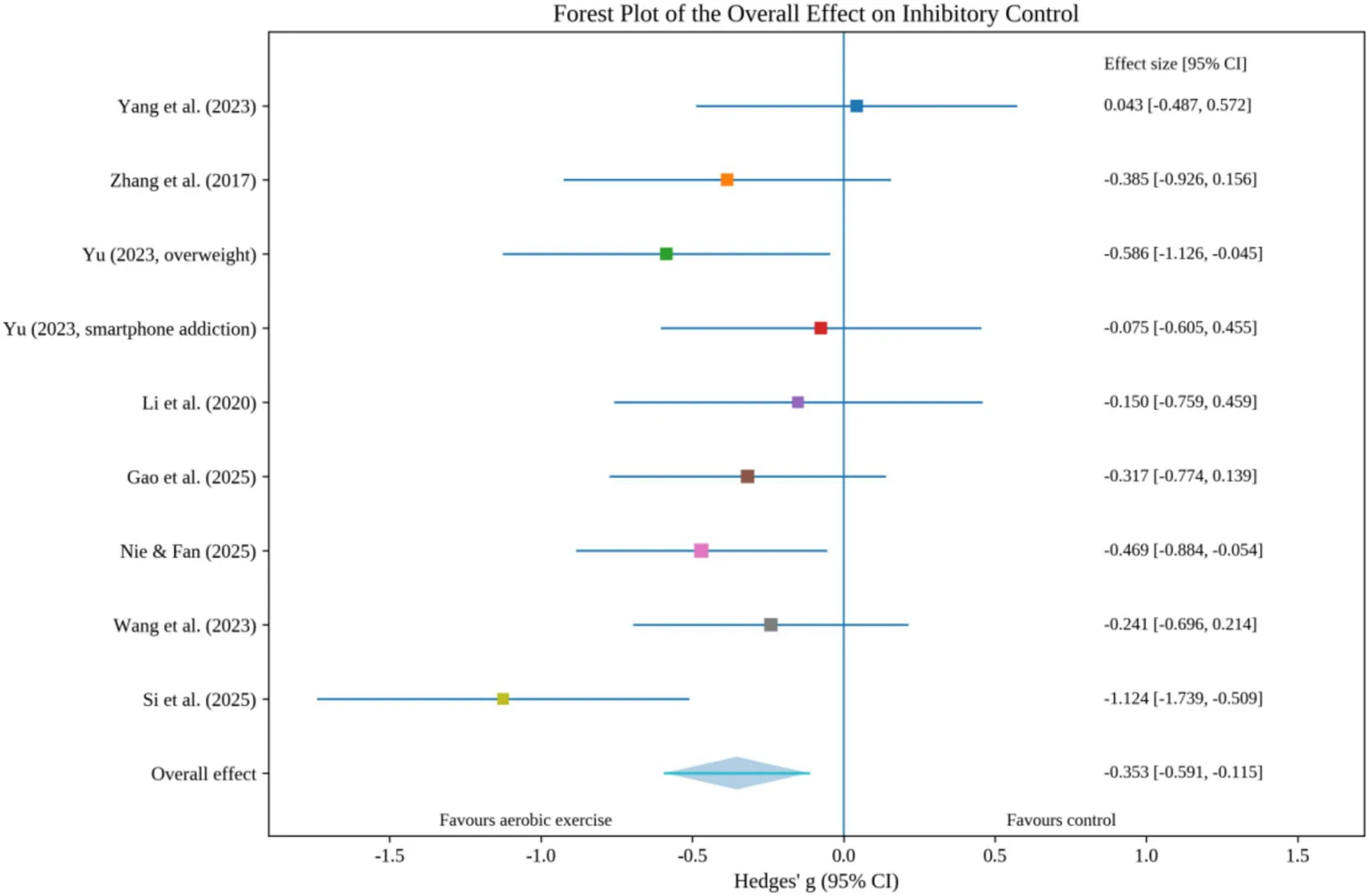
Forest plot of the overall effect of aerobic exercise on inhibitory control in university students. Note: Squares represent individual study effect estimates, horizontal lines represent 95% confidence intervals, and the diamond represents the pooled effect estimate. Negative Hedges’ *g* values favor aerobic exercise, whereas positive values favor the control condition.

### 3.3 Subgroup analysis of the effects of aerobic exercise interventions

Building on the existing research literature and the characteristics of the included studies, this study conducted exploratory subgroup analyses of factors that may influence the effects of aerobic exercise interventions. These primarily included participant type (general population versus special populations), intervention period (single exercise session versus long-term intervention), exercise intensity (moderate intensity versus high intensity), exercise session duration (≤30 min versus >30 min), and task paradigm (Stroop task versus Flanker task).

The results of the subgroup analysis showed that the direction of the effect was favourable to the aerobic exercise group across different participant types, intervention periods, exercise intensities, exercise session durations and task paradigms. The effect sizes for the general population and special populations were *g* = −0.353 and *g* = −0.371, respectively, and the difference between the groups was not statistically significant (*p* = 0.916); The effect sizes for single-session exercise and long-term intervention were *g* = −0.429 and *g* = −0.331, respectively, with no statistically significant difference between the groups (*p* = 0.698); The effect sizes for moderate-intensity and high-intensity exercise were *g* = −0.422 and *g* = −0.227, respectively; although the effect size in the moderate-intensity subgroup was numerically larger, the difference between groups was not statistically significant (*p* = 0.411); The effect sizes for the ≤30 min group and the >30 min group were *g* = −0.273 and *g* = −0.429, respectively; the difference between the groups was not statistically significant (*p* = 0.503); The effect sizes for the Flanker task and the Stroop task were *g* = −0.364 and *g* = −0.359, respectively; the difference between the groups was likewise not statistically significant (*p* = 0.978).

There were some differences in the level of heterogeneity across the various subgroups. Specifically, the *I*^2^ value for the general population subgroup was 54.71 per cent; the subgroups comprising long-term intervention, moderate-intensity exercise, exercise lasting >30 min and the Stroop task all exhibited a certain degree of between-study heterogeneity, whilst the subgroups comprising special populations, single-session exercise, high-intensity exercise, exercise lasting ≤30 min and the Flanker task showed relatively low levels of heterogeneity. Overall, none of the tests for between-group differences in the moderating factors reached statistical significance; given that some subgroups comprised a small number of studies, the relevant results should be interpreted with caution (see Table 3).

**Table 3:** Subgroup analyses of the effects of aerobic exercise on inhibitory control.

Subgroup	*k*	Hedges’ g (95% CI)	*t (df)*	*Q*_w_	*I*^2^ (%)	*τ^2^*	*p* (between)
Participant type							
General population	5	−0.353 (−0.890, 0.183)	−1.829 (4)	8.833	54.71	0.096	0.916
Special population	4	−0.371 (−0.693, −0.049)	−3.663 (3)	2.076	0.00	0.000	
Intervention period							
Single	2	−0.429 (−2.109, 1.251)	−3.244 (1)	0.553	0.00	0.000	0.698
Long-term	7	−0.331 (−0.662, 0.001)	−2.444 (6)	10.158	40.93	0.041	
Exercise intensity							
High intensity	6	−0.227 (−0.524, 0.071)	−1.961 (5)	3.065	0.00	0.000	0.411
Moderate intensity	11	−0.422 (−0.745, −0.099)	−2.912 (10)	17.279	42.13	0.076	
exercise session duration							
≤30 min	4	−0.273(−0.651, 0.105)	−2.299 (3)	2.704	0.00	0.000	0.503
>30 min	5	−0.429(−0.914, 0.056)	−2.458 (4)	7.494	46.63	0.061	
Task Paradigm							
Flanker	4	−0.364 (−0.778, 0.050)	−2.795 (3)	3.162	5.12	0.000	0.978
Stroop	5	−0.359 (−0.851, 0.134)	−2.023 (4)	7.778	48.58	0.065	

k denotes the number of independent studies or effect-size comparisons. For exercise intensity, k represents the number of intervention–control comparisons because separate intervention arms were retained. Hedges’ *g*, standardized effect size; CI, confidence interval; *t* (df), Hartung–Knapp-adjusted test statistic with corresponding degrees of freedom; Qw, within-subgroup heterogeneity statistic; *I*^2^, statistical heterogeneity; τ^2^, between-study variance estimated using REML; *p* (between), *p*-value for between-subgroup differences. Effect estimates, 95% CIs, t statistics, and degrees of freedom were derived from the same REML random-effects model with Hartung–Knapp adjustment. Negative Hedges’ g values favor aerobic exercise.

### 3.4 Exploratory univariate multilevel meta-regression analysis of the effects of aerobic exercise interventions

To further examine potential moderating factors, this study established a multilevel random-effects meta-regression model whilst retaining information on the different exercise intervention arms, with multiple effect sizes from the same study nested within the study. Given the limited number of included independent studies, and to minimise the risk of model overfitting and unstable parameter estimates that might arise from including multiple moderator variables simultaneously within a single model, this study established exploratory univariate multilevel meta-regression models using exercise intensity, intervention period, participant type, exercise session duration and task paradigm as individual moderator variables; the specific results are presented in Table 4.

**Table 4:** Exploratory univariable multilevel meta-regression analysis of potential moderators of aerobic exercise interventions.

Moderator	Comparison	Control	b (95% CI)	SE	*t*	*p*
Exercise intensity	High intensity	Moderate intensity	0.197 (−0.318 ~ 0.712)	0.218	0.906	0.395
Intervention period	Single	Long-term	−0.079 (−0.702 ~ 0.545)	0.264	−0.298	0.774
Participant type	Special population	General population	−0.019 (−0.548 ~ 0.510)	0.224	−0.085	0.935
exercise session duration	>30 min	≤30 min	−0.176 (−0.668 ~ 0.317)	0.208	−0.842	0.427
Task Paradigm	Stroop	Flanker	0.023 (−0.507 ~ 0.553)	0.224	0.104	0.920

Each model includes one moderator variable; multiple effect sizes from the same study are nested within the study. b denotes the meta-regression coefficient for the comparison level relative to the reference category. Moderate intensity, long-term intervention, general population, ≤30 min and the Flanker task were used as the reference categories for exercise intensity, intervention period, participant type, exercise session duration and task paradigm, respectively. b, meta-regression coefficient; SE, standard error; CI, confidence interval.

The meta-regression results showed that none of the five single-factor models revealed a statistically significant moderating effect. Using moderate intensity as the reference, the regression coefficient for high-intensity exercise was *b* = 0.197 (95% CI: −0.318 to 0.712, *p* = 0.395); with long-term intervention as the reference, the regression coefficient for a single exercise session was *b* = −0.079 (95% CI: −0.702 to 0.545, *p* = 0.774); with the general population as the reference, the regression coefficient for special population groups was *b* = −0.019 (95% CI: −0.548 to 0.510, *p* = 0.935); Using exercise session duration of 30 min or less as the reference, the regression coefficient for exercise sessions lasting longer than 30 min was *b* = −0.176 (95% CI: −0.668 to 0.317, *p* = 0.427); Taking the Flanker task as the reference, the regression coefficient for the Stroop task was *b* = 0.023 (95% CI: −0.507 to 0.553, *p* = 0.920).

Overall, no statistically significant moderating effects were found in any of the univariable meta-regression models. Given that this study included only nine independent studies, the statistical power of the meta-regression analyses and the precision of the parameter estimates remain limited; therefore, the above results are primarily intended to explore potential moderating factors, and non-significant results should not be interpreted as indicating that the factors in question do not exert a moderating effect.

## 4 Discussion

This meta-analysis synthesised evidence from randomised controlled trials examining the effects of aerobic exercise on inhibitory control in university students. Compared with no exercise intervention, routine activity or a waiting list control, aerobic exercise demonstrated a small but statistically significant improvement in inhibitory control among university students (*g* = −0.353, 95% CI: −0.591 to −0.115, *p* = 0.009). However, this effect is generally of modest magnitude, and as existing studies have primarily utilised laboratory-based inhibitory control tasks such as the Stroop and Flanker tests for evaluation, it remains to be verified whether this can be further translated into practical improvements in learning, self-regulation or other aspects of daily functioning. The statistical heterogeneity of the overall effect size was low, and neither the direction of the effect nor its statistical significance changed when individual studies were excluded one by one, indicating that the overall results possess a certain degree of stability. At the same time, the low *I*^2^ value merely reflects the relatively limited statistical heterogeneity observed among the effect sizes; it does not imply that the included studies are highly homogeneous in terms of clinical and methodological characteristics. Overall, this study focuses specifically on the executive function component of inhibitory control in university students and explores whether exercise intensity, intervention period, exercise session duration, participant type and task paradigm are associated with variability in the intervention effects.

Previous research generally supports the notion that aerobic exercise has a certain promotional effect on executive function; however, findings regarding inhibitory control have not been entirely consistent (Chang et al., 2025; Ciria et al., 2018; Kao et al., 2022; Remiszewski et al., 2025). Some studies have found that both single-session and long-term aerobic exercise can improve performance in conflict processing and response inhibition, whilst others have not observed consistent effects (Ciria et al., 2018; Remiszewski et al., 2025). These discrepancies may be related to the use of varying exercise intensities, modes of exercise and inhibitory tasks across previous studies (Aziz-Safaie et al., 2024; Bailey et al., 2021; Youssef et al., 2024). Building upon these studies, the present study further demonstrates, through quantitative meta-analysis that aerobic exercise has an overall small-effect-size improvement on inhibitory control in university students, thereby providing comprehensive evidence to reconcile the inconsistent findings observed in previous individual studies. It should be noted that this effect reflects the overall average results of different aerobic exercise programmes, rather than a fixed effect of any specific exercise programme. Furthermore, single acute exercise sessions and long-term exercise training occur on different time scales, and their effects on inhibitory control may involve different physiological and cognitive processes. Therefore, the overall pooled effect in this study should be interpreted as the average effect across different forms of aerobic exercise exposure, rather than implying that acute exercise and long-term training share the same mechanism of action or pattern of effects. Upon further analysis restricting the inclusion to studies on long-term exercise training alone, the direction and magnitude of the effect were similar to those of the overall analysis, although the confidence interval extended slightly across 0, suggesting that the overall effect was not clearly driven by studies on acute exercise, whilst the precision of the evidence from long-term training remains limited.

The potential mechanisms by which aerobic exercise improves inhibitory control may involve exercise-induced short-term physiological changes and long-term neural adaptations (Youssef et al., 2024). Previous research suggests that aerobic exercise may be associated with changes in physiological arousal, cerebral haemodynamics, neurotrophic factors and prefrontal-related neural activity, thereby providing a potential neurophysiological explanation for the relationship between aerobic exercise and inhibitory control (Bailey et al., 2021; Ludyga et al., 2024). It should be emphasised that the outcomes used for effect size pooling in this meta-analysis were all measures from behavioural tasks such as the Stroop and Flanker tests; no quantitative pooling was performed for neurophysiological indicators such as cerebral blood flow, neurotrophic factors, or EEG, fNIRS and fMRI. Consequently, the mechanisms described above serve merely as theoretical explanations based on previous research, rather than findings directly supported by this meta-analysis; the relevant mechanisms still require further validation in subsequent studies.

Exploratory moderation analyses indicated that exercise intensity, intervention period, exercise session duration, participant type and task paradigm all showed no statistically significant between-group differences. Notably, moderate-intensity exercise showed a numerically larger effect estimate than high-intensity exercise; this is consistent to some extent with findings from some previous studies suggesting that moderate-intensity aerobic exercise may be more beneficial for inhibitory control (Chang et al., 2025). Previous research has suggested that one possible explanation is that moderate-intensity exercise may enhance physiological arousal and cognitive readiness, whilst avoiding the fatigue and competition for resources associated with higher-intensity exercise (Bailey et al., 2021). However, as neither the differences between the intensity subgroups nor the results of the meta-regression reached statistical significance, the current evidence is insufficient to confirm that moderate-intensity exercise is superior to high-intensity exercise, let alone to establish a consistent U-shaped dose–response relationship. Similarly, longer exercise session duration showed a certain advantage in terms of effect size, but the differences between groups were not significant, suggesting that the effects of exercise intensity and duration may be jointly influenced by factors such as exercise mode, total workload and the timing of testing (Chang et al., 2025).

Participant type, intervention period and task paradigm likewise showed no statistically significant between-group differences. Both special populations and general university students exhibited an effect direction favourable to exercise, and the pooled estimates for single-session exercise and long-term interventions were also in the same direction. However, given the limited number of studies within each subgroup, these non-significant differences should not be interpreted as evidence that the effects were equivalent across participant groups or intervention periods, or that these factors do not moderate the intervention effect. The special populations themselves also encompassed diverse characteristics, including overweight status, internet or smartphone addiction and depressive symptoms. The effect sizes obtained from the Flanker and Stroop tasks were numerically similar; however, this should likewise not be interpreted as evidence of equivalent effects across task paradigms, particularly given the differences between these paradigms in stimulus characteristics, conflict processing and response demands (Aziz-Safaie et al., 2024). A further exploratory univariable meta-regression also did not identify any statistically significant moderating associations; given the limited number of independent studies, these findings should be regarded as exploratory and interpreted with caution. Consequently, the current evidence is better suited to describing trends in potential effects than to determining the presence or absence of specific moderating effects or identifying the optimal exercise programme.

Certain limitations should be acknowledged. Firstly, the number of independent studies included was limited, and none were assessed as having an overall low risk of bias; two of the studies were classified as having a high risk of bias, which limits the certainty of the conclusions. Furthermore, all nine included studies were conducted in China; therefore, the current findings primarily reflect a sample of Chinese university students, and caution should be exercised when generalising the results to university student populations with different cultural, educational and geographical backgrounds. Secondly, this study restricted the participants to university students aged 18–25, with the aim of focusing on a relatively consistent developmental stage and reducing the heterogeneity that a wider age range might entail. However, the age composition of university students may vary across different countries and educational systems, and some students may be over 25 years of age; therefore, the applicability of the current findings to older university student populations requires further validation. Thirdly, there are few long-term follow-up studies; the existing evidence mainly reflects the effects of acute or short-term interventions, and the long-term maintenance of the benefits of aerobic exercise on inhibitory control remains unclear. Fourthly, some studies employed multi-arm designs, and differences between studies in terms of exercise intensity, duration and intervention methods have increased the complexity of identifying moderating effects. Furthermore, as pooled analyses and moderating analyses retain information at different levels of detail, direct numerical comparisons of the corresponding estimates are not appropriate. Fifth, some subgroups comprise only a small number of studies, limiting the statistical power of between-group comparisons. Subgroups of specific populations include samples with varying characteristics—such as overweight individuals, those with internet or smartphone addiction, and those with depressive symptoms—resulting in considerable within-group heterogeneity; therefore, the relevant findings should be interpreted with caution. Sixth, exploratory single-factor meta-regression remains limited in terms of statistical power and estimation accuracy due to the limited number of independent studies. Therefore, future research should include more high-quality, large-sample randomised controlled trials; conduct direct comparisons of different intensities and durations whilst standardising exercise load and measures of inhibitory control; and incorporate long-term follow-up and neurophysiological indicators to further clarify the characteristics and potential mechanisms by which aerobic exercise improves inhibitory control in university students.

## 5 Concllusion

Although the certainty of the existing evidence remains limited, the results of this meta-analysis suggest that aerobic exercise may lead to a modest improvement in university students’ performance on inhibitory control tasks; however, the practical significance of this effect remains unclear, and the representativeness and generalisability of the current evidence are still subject to certain limitations. Exploratory analyses did not reveal any statistically significant moderating associations between the intervention effect and participant type, intervention period, exercise intensity, exercise session duration, or task paradigm, although some subgroups exhibited certain differences in effect sizes. Given the limited number of independent studies, these non-significant findings should not be interpreted as evidence of equivalent effects across subgroups or the absence of moderation. Overall, the available evidence provides preliminary quantitative support for the notion that aerobic exercise may improve inhibitory control in university students; however, these conclusions require further validation through additional high-quality studies to clarify the roles of different exercise parameters and their dose–response relationships.

## Data Availability

The original contributions presented in the study are included in the article/Supplementary material, further inquiries can be directed to the corresponding author/s.
