# Supplementary material for: A meta-analysis of the effects of aerobic exercise on inhibitory control in university students and an analysis of moderating effects
Source: Front Psychol. 2026 Oct 29;17:1992560. doi: 10.3389/fpsyg.2026.1992560 (PMC13755330; doi:10.3389/fpsyg.2026.1992560)
Supplement: Supplementary file 1 [file Supplementary_file_1.docx]

**Pubmed=2349**

Search terms：("Students"[Mesh] OR "Young Adult"[Mesh] OR college student[Title/Abstract] OR university student[Title/Abstract] OR undergraduate[Title/Abstract]) AND ("Exercise"[Mesh] OR "Aerobic Exercise"[Mesh] OR aerobic[Title/Abstract] OR endurance[Title/Abstract] OR running[Title/Abstract] OR cycling[Title/Abstract] OR swimming[Title/Abstract] OR walking[Title/Abstract]) AND ("Cognition"[Mesh] OR "Memory"[Mesh] OR "Attention"[Mesh] OR "Executive Function"[Mesh] OR cognit[Title/Abstract] OR memory[Title/Abstract] OR attention[Title/Abstract] OR "executive function"[Title/Abstract])NOT ("Child"[Mesh] OR "Adolescent"[Mesh] OR "Aged"[Mesh] OR child[Title/Abstract] OR adolescent[Title/Abstract] OR elderly[Title/Abstract])

**Web of science=3068**

Search terms：TS=("students" OR "young adult" OR "college student" OR "university student" OR "undergraduate") AND TS=("exercise" OR "aerobic exercise" OR "aerobic" OR "endurance" OR "running" OR "cycling" OR "swimming" OR "walking") AND TS=("cognition" OR "memory" OR "attention" OR "executive function" OR "cognit") NOT TS=("child" OR "adolescent" OR "aged" OR "elderly")

**APA PsycArticles=20**

Search terms：SU ("students" OR "young adult" OR "college student" OR "university student" OR "undergraduate") AND ("exercise" OR "aerobic exercise" OR "aerobic" OR "endurance" OR "running" OR "cycling" OR "swimming" OR "walking") AND ("cognition" OR "memory" OR "attention" OR "executive function" OR "cognit) NOT SU ("child" OR "adolescent" OR "aged" OR "elderly")

**APA Psycinfo=408**

Search terms：SU ("students" OR "young adult" OR "college student" OR "university student" OR "undergraduate") AND ("exercise" OR "aerobic exercise" OR "aerobic" OR "endurance" OR "running" OR "cycling" OR "swimming" OR "walking") AND ("cognition" OR "memory" OR "attention" OR "executive function" OR "cognit) NOT SU ("child" OR "adolescent" OR "aged" OR "elderly")

**Cochrane=1486**

Search terms：

#1	MeSH descriptor: [Students] explode all trees	9255

#2	(students):ti,ab,kw OR ("university student"):ti,ab,kw OR ("young adult"):ti,ab,kw OR (college students):ti,ab,kw OR ("undergraduate"):ti,ab,kw	156653

#3	#1 OR #2	156680

#4	MeSH descriptor: [Cognition] explode all trees	17148

#5	("cognition"):ti,ab,kw OR (memory):ti,ab,kw OR (attention):ti,ab,kw OR (executive function):ti,ab,kw OR (cognit):ti,ab,kw	101130

#6	#4 OR #5	104254

#7	MeSH descriptor: [Exercise] explode all trees	42085

#8	("exercise"):ti,ab,kw OR ("aerobic exercise"):ti,ab,kw OR (endurance):ti,ab,kw OR (running):ti,ab,kw OR (walking):ti,ab,kw	177680

#9	#7 OR #8	179650

#10	#3 and #6 #9	1489

**Elsevier=1204**

Search terms：TITLE-ABS-KEY("students" OR "young adult" OR "college student" OR "university student" OR "undergraduate") AND TITLE-ABS-KEY("exercise" OR "aerobic exercise" OR "aerobic" OR "endurance" OR "running" OR "cycling" OR "swimming" OR "walking") AND TITLE-ABS-KEY("cognition" OR "memory" OR "attention" OR "executive function" OR "cognit*") AND NOT TITLE-ABS-KEY("child" OR "aged" OR "elderly")

**CNKI=246**

Search terms：(主题: 学生 OR 大学生 OR 青年 OR 本科生 OR 高校学生) AND (主题: 有氧运动 OR 有氧训练 OR 运动干预 OR 耐力训练 OR 骑行 OR 慢跑 OR 步行 OR 游泳) AND (主题: 认知功能 OR 认知能力 OR 执行功能 OR 注意力 OR 记忆力 OR 抑制控制)

NOT (主题: 老年人 OR 儿童 OR 未成年)

**WanFang=1418**

Search terms：主题:(学生 or 大学生 or 青年 or 本科生 or 高校学生) and 主题:(有氧运动 or 有氧训练 or 运动干预 or 耐力训练 or 骑行 or 慢跑 or 步行 or 游泳) and 主题:(认知功能 or 认知能力 or 执行功能 or 抑制控制 or 注意力 or 记忆力) not 主题:(老年人 or 儿童 or 未成年)

**VIP=47**

Search terms:(M=学生 OR M=大学生 OR M=青年 OR M=本科生 OR M=高校学生) AND (M=有氧运动 OR M=有氧训练 OR M=运动干预 OR M=耐力训练 OR M=骑行 OR M=慢跑 OR M=步行 OR M=游泳) AND (M=认知功能 OR M=认知能力 OR M=执行功能 OR M=注意力 OR M=记忆力 OR M=抑制控制) NOT (M=老年人 OR M=儿童 OR M=未成年)

**Total：10246**
