# Supplementary material for: A meta-analysis of the effects of aerobic exercise on inhibitory control in university students and an analysis of moderating effects
Source: Front Psychol. 2026 Oct 29;17:1992560. doi: 10.3389/fpsyg.2026.1992560 (PMC13755330; doi:10.3389/fpsyg.2026.1992560)
Supplement: Supplementary file 2 [file Table_1.docx]

**Supplementary Table S1. Basis for the Extraction and Calculation of Effect Sizes in the Study**

| **Study** | **Task paradigm and outcome metric** | **Intervention post-test data: M±SD (n)** | **Combined intervention: M±SD (n)** | **Control post-test data: M±SD (n)** | **Direction harmonization** | **Hedges’ g** | **95% CI** |
| --- | --- | --- | --- | --- | --- | --- | --- |
| Yang et al. (2023) | Flanker; interference score (incongruent − congruent) | A: −7.32±46.08 (n=20) B: 3.78±35.61 (n=20) | −1.77±41.03 (n=40) | −3.57±42.95 (n=20) | Direction harmonized according to the original metric; negative g favors exercise | 0.043 | −0.487 to 0.572 |
| Zhang et al. (2017) | Flanker; interference score (incongruent − congruent) | 1.22±12.04 (n=26) | 1.22±12.04 (n=26) | 5.58±10.20 (n=26) | Direction harmonized according to the original metric; negative g favors exercise | −0.385 | −0.926 to 0.156 |
| Yu (2023, overweight college students) | Flanker; incongruent-trial reaction time | A1: 530.87±41.11 (n=20) B1: 495.07±35.23 (n=20) | 512.97±41.91 (n=40) | 538.86±46.98 (n=20) | Lower reaction time = better performance; no sign reversal | −0.586 | −1.126 to −0.045 |
| Yu (2023, college students with smartphone addiction) | Stroop; incongruent-trial reaction time | A2: 924.24±105.09 (n=20) B2: 861.67±39.42 (n=20) | 892.96±84.51 (n=40) | 906.23±281.61 (n=20) | Lower reaction time = better performance; no sign reversal | −0.075 | −0.605 to 0.455 |
| Li et al. (2020) | Stroop; incongruent-trial reaction time | 995.98±184.37 (n=21) | 995.98±184.37 (n=21) | 1022.00±151.71 (n=19) | Lower reaction time = better performance; no sign reversal | −0.150 | −0.759 to 0.459 |
| Gao et al. (2025) | Stroop; incongruent-trial reaction time | A: 638.00±43.00 (n=27) B: 574.00±73.00 (n=26) | 606.60±67.30 (n=53) | 627.00±56.00 (n=28) | Lower reaction time = better performance; no sign reversal | −0.317 | −0.774 to 0.139 |
| Nie & Fan (2025) | Flanker; interference score (incongruent − congruent) | A: −25.60±35.92 (n=30) B: −22.60±58.89 (n=30) C: −46.60±43.77 (n=30) | −31.60±47.85 (n=90) | −56.60±66.11 (n=30) | Sign reversed during direction harmonization so that negative g favors exercise | −0.469 | −0.884 to −0.054 |
| Wang et al. (2023) | Stroop; incongruent-trial reaction time | A: 628.50±164.52 (n=27) B: 599.00±161.13 (n=26) | 614.03±161.98 (n=53) | 653.88±167.84 (n=28) | Lower reaction time = better performance; no sign reversal | −0.241 | −0.696 to 0.214 |
| Si et al. (2025) | Stroop; overall outcome | A: 692.38±123.71 (n=17) B: 643.34±99.24 (n=17) | 667.86±113.20 (n=34) | 786.07±79.89 (n=17) | Direction harmonized according to the original metric; negative g favors exercise | −1.124 | −1.739 to −0.509 |

Note. The primary analysis effect size was calculated based on between-group comparisons following the end of the intervention. For multi-arm randomised trials, the primary analysis pooled the intervention arms that met the inclusion criteria and compared them with a shared control group. After standardising the direction of the effect size, a value of Hedges’ g < 0 indicates that the aerobic exercise group was more effective at suppressing control behaviour than the control group. M, mean; SD, standard deviation; CI, confidence interval.
